# SIRT1 alleviates senescence of degenerative human intervertebral disc cartilage endo-plate cells via the p53/p21 pathway

**DOI:** 10.1038/srep22628

**Published:** 2016-03-04

**Authors:** Nian Zhou, Xin Lin, Wen Dong, Wei Huang, Wei Jiang, Liangbo Lin, Quanhe Qiu, Xiaojun Zhang, Jieliang Shen, Zhaojun Song, Xi Liang, Jie Hao, Dawu Wang, Zhenming Hu

**Affiliations:** 1 Department of Orthopaedic Surgery, the First Affiliated Hospital of Chongqing Medical University, Chongqing, China; 2 Department of Rehabilitation Medicine, the First Affiliated Hospital of Chongqing Medical University, Chongqing, China

## Abstract

Cartilage end plates (CEP) degeneration plays an integral role in intervertebral disc (IVD) degeneration resulting from nutrient diffusion disorders. Although cell senescence resulting from oxidative stress is known to contribute to degeneration, no studies concerning the role of senescence in CEP degeneration have been conducted. SIRT1 is a longevity gene that plays a pivotal role in many cellular functions, including cell senescence. Therefore, the aim of this study was to investigate whether senescence is more prominent in human degenerative CEP and whether SIRT1-regulated CEP cells senescence in degenerative IVD as well as identify the signaling pathways that control that cell fate decision. In this study, the cell senescence phenotype was found to be more prominent in the CEP cells obtained from disc degenerative disease (DDD) patients than in the CEP cells obtained from age-matched lumbar vertebral fractures (LVF) patients. In addition, the results indicated that p53/p21 pathway plays an important role in the senescence of CEP cells *in vivo* and *vitro*. Furthermore, SIRT1 was found to be capable of alleviating the oxidative stress-induced senescence of CEP cells in humans via p53/p21 pathway. Thus, the information presented in this study could be used to further investigate the underlying mechanisms of CEP.

Intervertebral disc (IVD) degeneration disorders are associated with high morbidity rates and decreased quality of life^1^. However, the underlying mechanisms of IVD degeneration are not well understood. IVD consists of a nucleus pulposus (NP), an annulus fibrosus (AF), and cartilage end plates (CEP). Excluding the sparse blood supply in the outer layers of the annulus, mature discs rely almost entirely on the diffusion of solutes across the cartilage end-plates for nutrients^2^,^3^. Therefore, since CEP degeneration likely impedes the diffusion of nutrients between vertebral marrow and discs, accelerating IVD degeneration, investigating the underlying mechanisms of CEP degeneration is integral to understanding IVD degeneration^2^,^4^.

Silent information regulation 2 (Sir2), an NAD-dependent deacetylase, is associated with life span development^5^. Sir2 is capable of extending the life span of organisms such as yeast and flies^5^. Recent studies have shown that silent information regulation 2 homolog-1 (SIRT1), the equivalent of Sir2 yeast in mammalian cells, is a longevity gene that plays a pivotal role in many cellular functions, including cell differentiation and proliferation, apoptosis, and cell aging^6^. In our previous studies, SIRT1 expression was found to be reduced in degenerative NP tissue, and SIRT1 was found to inhibit the apoptosis of degenerative NP cells in human discs^7^,^8^.

Cell senescence, which occurs when normal cells stop dividing, is thought to be a contributor to IVD degeneration^9^,^10^. Senescent biomarkers increase significantly with increasing degrees of disc degeneration^10^. Because senescent cells cannot divide, senescence can reduce the ability of discs to generate new cells and, thereby, replace those lost as a result of necrosis or apoptosis^11^. Although the underlying mechanisms of senescence pertaining to IVD degeneration in humans are unclear, oxidative stress has been proven to play a primary role in the regulation of senescence and degeneration^9^. In addition, the relationship between oxidative stress and SIRT1 activity has been theorized to be integral to the aging process^12^. However, until recently, no studies concerning the role of senescence in CEP degeneration have been conducted^3^, let alone the role of SIRT1 in CEP cell senescence.

The aim of this study was to determine the relative cell senescence phenotype expression of CEP cells obtained from patients with lumbar disc degenerative disease (DDD) and age-matched patients with lumbar vertebral fractures (LVF). In addition, the regulatory mechanisms and signaling pathways of SIRT1 in CEP cells undergoing oxidative stress-induced senescence were investigated.

## Results

### Cell senescence phenotype expression in the CEP cells of the DDD and LVF patients

**Figure 1:**
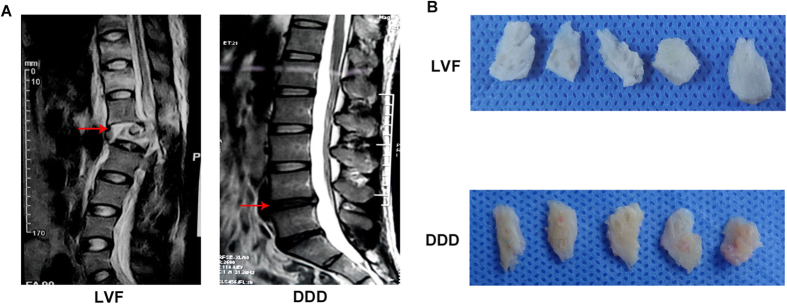
Representative MRIs and CEP samples. (**A**) MRI scans of spines with LVF and DDD. Featured left is a patient with lumbar LVF undergoing posterior discectomy, spinal fusion, decompression, and stability within 24 hours of trauma. This patient had no formerly documented clinical history of low back pain (LBP). Featured right is a patient with DDD undergoing discectomy and intervertebral fusion surgery. The red arrow indicates the position of the experimental material. (**B**) CEP samples carefully isolated from IVD tissue with a scalpel.

**Figure 2:**
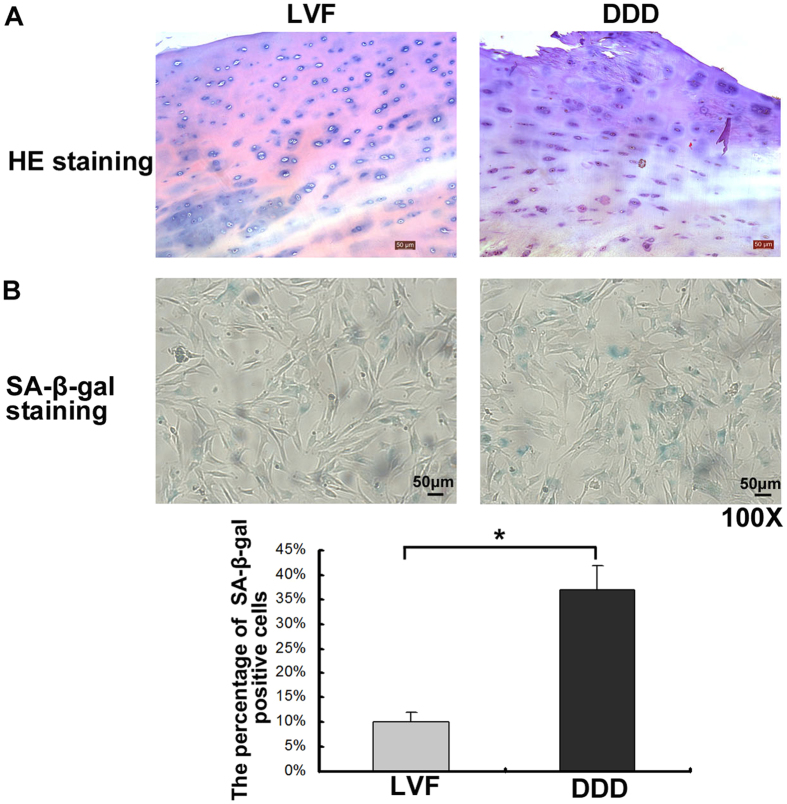
HE and SA-β-galactosidase staining of CEP samples (100X). (**A**) HE stainings of CEP samples obtained from patients with IVF and DDD. (**B**) SA-β-galactosidase stainings of CEP cells obtained from patients with LVF and DDD (100X). (**C**) The number of SA-β-Gal-positive cells (blue) and staining intensity of the CEP cells obtained from all the DDD patients were significantly greater than those of the CEP cells obtained from all the LVF patients. Three independent experiments were performed on the tissues from three patients for each group, and the data was denoted in terms of mean ± SD. *P < 0.05. NS: *P* > 0.05.

Representative MRI scans of spines with IVF and DDD were shown in Fig. 1A. CEP samples were collected, as shown in Fig. 1B. The samples obtained from the all the DDD patients were yellow in color and exhibited varying levels of calcification according to their macroscopic morphologies. As shown in the HE staining (Fig. 2A), the borders of the CEP cells obtained from all the IVF patients were rounded, while the borders of the CEP cells obtained from the all DDD patients were platy. In addition, as shown in the SA-β-Gal staining (Fig. 2B,C), both the percentage of SA-β-Gal-positive cells (blue) and staining intensity of the CEP cells obtained from all the DDD patients were significantly greater than those of the CEP cells obtained from all the LVF patients.

**Figure 3:**
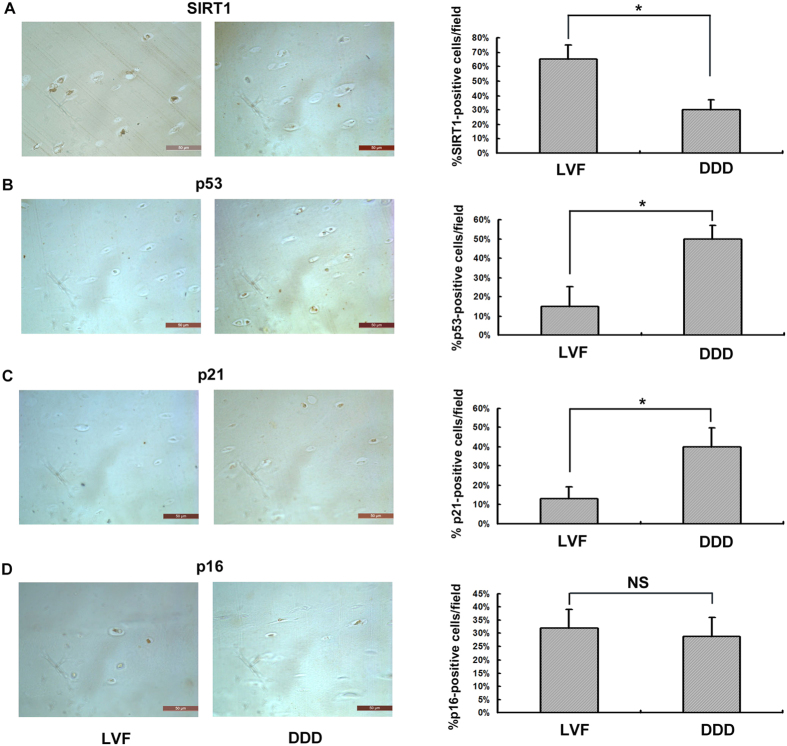
Immunohistochemical examination of SIRT1 and cellular senescence pathway expression in degenerative human CEP samples. (**A**) Reduced SIRT1 expression in the patient with DDD (100X, *P < 0.05). (**B**) Increased p53 expression in the patient with DDD. (**C**) Increased p21 expression in the patient with DDD. (**D**) Insignificant reduction in p16 expression in the patient with DDD. Three independent experiments were performed on the tissues from three patients for each group, and the data was denoted in terms of mean ± SD. *P < 0.05. NS: *P* > 0.05.

As shown in Fig. 3A, the immunohistochemical detection results indicated that the CEP samples obtained from the DDD patients exhibited a significantly lower number of SIRT1-postive cells than the CEP samples obtained from the LVF patients (*p* < 0.05). In contrast, as shown in Fig. 3B,C, a higher number of p53- and p21-positive cells was observed in the DDD samples than in the LVF samples (*p* < 0.05). No significant differences in the number of p16-positive cells in the LVF and DDD groups existed (*p* > 0.05) (Fig. 3D).

### Effects of oxidative stress on CEP cell senescence

**Figure 4:**
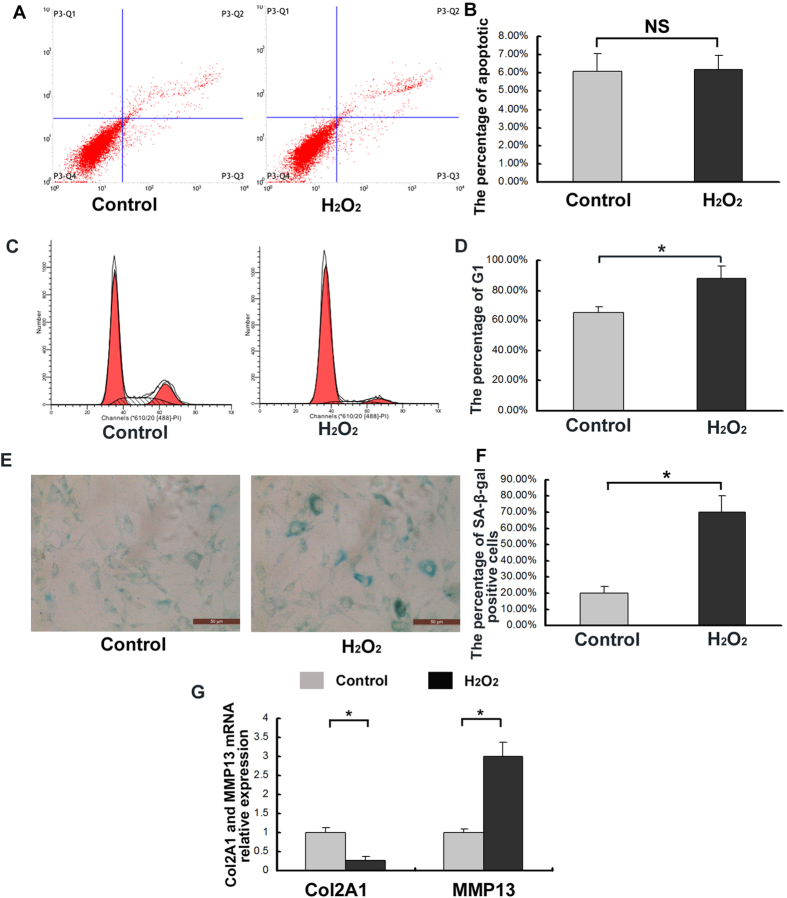
Increased levels of degenerative human CEP cell senescence resulting from oxidative stress induced by 150 μM H_2_O_2_. (**A**) Sublethal oxidative stress induced by H_2_O_2_, and the early-and late-apoptosis of CEP cells were detected with Flow cytometry. (**B**) The addition of H_2_O_2_ did not increase CEP cell apoptosis. (**C**) Sublethal oxidative stress induced by H_2_O_2_, and the cells in different cycles including G_1_, G_2_ and S were counted and represented as a percentage of the total cell count with Flow cytometry. (**D**) The addition of H_2_O_2_ increased the population of G_1_ phase cells. (**E**) Sublethal oxidative stress induced by H_2_O_2_, and CEP cells were detected with SA-β-Gal staining. (**F**) The addition of H_2_O_2_ increased the percentage of SA-β-Gal-positive cells (blue) and the staining intensity. (**G**) The addition of H_2_O_2_ decreased Col2A1 expression and increased MMP13 mRNA expression. Three independent experiments were performed on the degenerative human CEP cells from three patients, and the data was denoted in terms of mean ± SD. *P < 0.05. NS: *P* > 0.05.

As shown in Fig. 4A,B, the sublethal oxidative stress induced by H_2_O_2_ did not cause increased levels of the early- and late-stage apoptosis in the CEP cells (*p* > 0.05). However, the H_2_O_2_-induced oxidative stress did significantly increase the population of G_1_ phase cells (*p* < 0.05) (Fig. 4C,D), and the percentage SA-β-Gal-positive cells (blue), and staining intensity (Fig. 4E,F). The expression of collagen II (Col2A1) was decreased and matrix metalloproteinase 13 (MMP13) was increased in oxidative stress (*p* < 0.05) (Fig. 4G).

### Effects of oxidative stress on the p53/p21 pathway of CEP cell senescence

**Figure 5:**
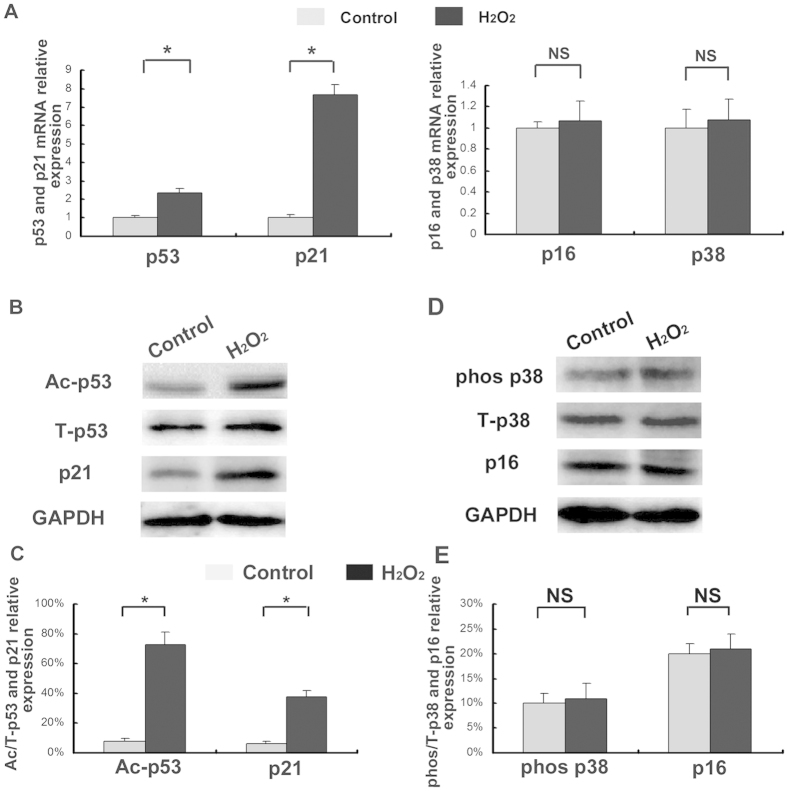
Activation of the p53/p21 pathway resulting from oxidative stress induced by 150 μM H_2_O_2_. (**A**) The addition of H_2_O_2_ increased p53 and p21 expression at the mRNA level, but did not increase p38 or p16 expression at the mRNA level. (**B**) Ac-p53, T-p53 and p21 protein expression were detected by Western blotting with H_2_O_2_ or not. (**C**) The addition of H_2_O_2_ increased the acetylated p53 (lys382) and p21 protein expression level. (**D**) phos-p38, T-p38 and p16 protein expression were detected by Western blotting with H_2_O_2_ or not. (**E**) The addition of H_2_O_2_ did not increase phosphorylated p38 or p16 protein expression level. The gels have been run under the same experimental conditions. Three independent experiments were performed on the degenerative human CEP cell from three patients, and the data was denoted in terms of mean ± SD. *P < 0.05. NS: *P* > 0.05.

Cellular senescence primarily occurs via two pathways, including the p38 mitogen activated protein kinase (MAPK)/p16^INK4a^ pathway and the p53/p21^cip^ pathway. Significant differences in the p53 mRNA levels were observed under oxidative stress (*p* < 0.05) (Fig. 5A). In addition, the levels of p21 RNA (Fig. 5A) and protein (Fig. 5B,C), pro-senescence modulators of p53 activity, increased significantly under H_2_O_2_-induced oxidative stress (*p* < 0.05). The levels of acetylated p53 were also higher under oxidative stress (Fig. 5B,C). In contrast, there was no significant increase for p38 and p16 levels of mRNA (*p* > 0.05) (Fig. 5A) and levels of phosphorylated p38 and p16 level of protein under oxidative stress (Fig. 5D,E).

### Effects of SIRT1 on the p53/p21 pathway and CEP cell senescence under oxidative stress

**Figure 6:**
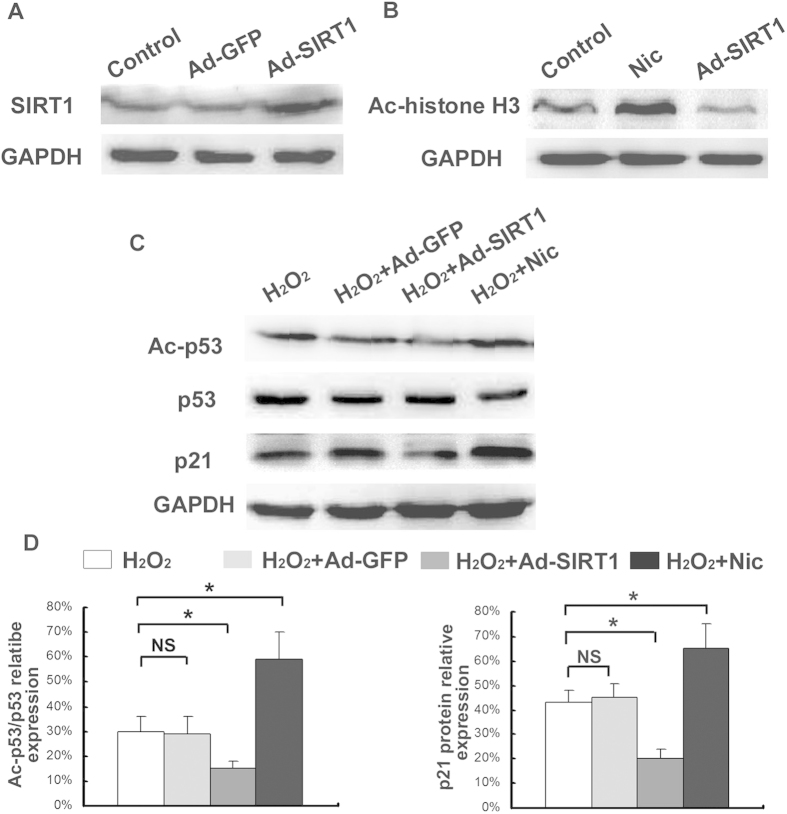
Inhibition of the p53/p21 pathway by SIRT1 in oxdative stress. (**A**) Western blot demonstrating the effectiveness of the Ad-SIRT1 virus in the overexpression of SIRT1. (**B**) Acetylated histone H_3_ levels analyzed via the Western blot method. (**C**) Overexpression of SIRT1 and decreased SIRT1 activity resulting from the addition of Ad-SIRT1 and nicotinamide, respectively. Acetylated p53 (lys382) and p21 levels analyzed via the Western blot method. (**D**) Percentage of relative Ac-p53/p53 and p21 protein expression. All of the relative protein expression levels were compared to the GAPDH using Quantity one. The gels have been run under the same experimental conditions. Three independent experiments were performed on the cells from three patients, and the data was denoted in terms of mean ± SD. Nic: nicotinamide. *P < 0.05. NS: *P* > 0.05.

**Figure 7:**
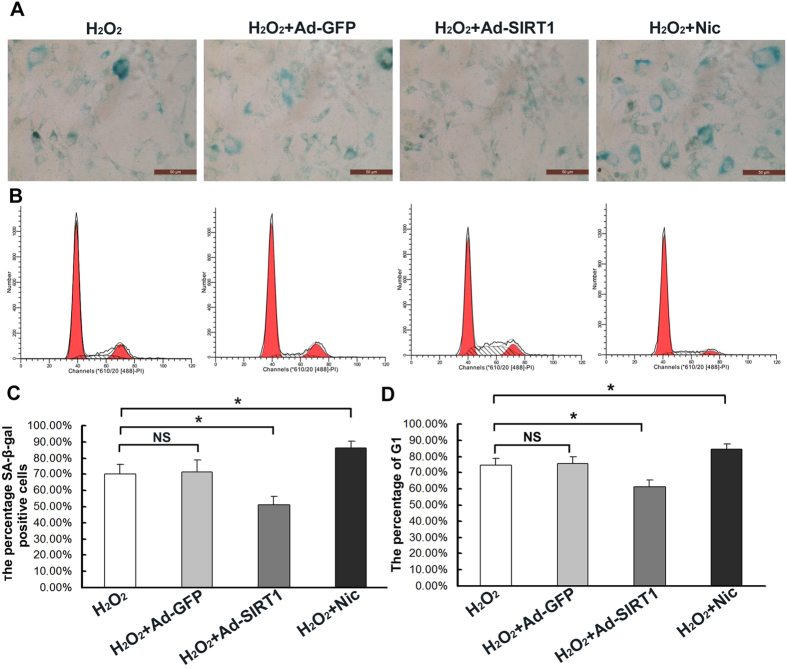
Decrease in the number of cells positive for SA-β-galactosidase and the population of G1 phase cells resulting from the overexpression of SIRT1 in oxdative stress. (**A**) The CEP SA-β-galactosidase staining was performed. (**B**) The CEP cell cycle detected with Flow cytometry. (**C**) The overexpression of SIRT1 decreased the number of cells positive for SA-β-galactosidase (blue) and the staining intensity, but the inhibition of SIRT1 activity with nicotinamide increased the number of cells positive for SA-β-galactosidase. (**D**) The overexpression of SIRT1 decreased the population of G_1_ phase cells, but the inhibition of SIRT1 activity with nicotinamide increased the population of G_1_ phase cells. Three independent experiments were performed on the degenerative human CEP cells from three patients, and the data was denoted in terms of mean ± SD. Nic: nicotinamide. *P < 0.05. NS: *P* > 0.05.

Oxidative stress and SIRT1 activity play an integral role in the regulation of the aging process. In order to determine whether SIRT1 influences oxidative stress-induced senescence, SIRT1 was overexpressed in the CEP cells via adenoviral-mediated gene delivery. The addition of Ad-SIRT1 resulted in increased SIRT1 expression (Fig. 6A) and decreased levels of acetylated histone-H_3_ (Fig. 6B). The addition of nicotinamide, which was used to decrease SIRT1 activity, significantly increased the levels of acetylated histone-H_3_ (Fig. 6B). Under sublethal oxidative stress, the overexpression of SIRT1 significantly decreased the levels of acetylated p53 as well as the levels of its pro-senescence effector p21 (*p* < 0.05) (Fig. 6C,D). The overexpression of SIRT1 also decreased the population of G_1_ phase cells (*p* < 0.05) (Fig. 7B,D), the percentege of SA-β-Gal-positive cells (blue), and staining intensity (Fig. 7A,C) of the CEP cells. In contrast, the addition of nicotinamide significantly increased the levels of acetylated p53 as well as the levels of its pro-senescence effector p21 (*p* < 0.05) (Fig. 6C,D). The nicotinamide also increased the population of G_1_ phase cells(*p* < 0.05) (Fig. 7B,D), the percentege of SA-β-Gal-positive cells (blue), and staining intensity (Fig. 7A,C) of the CEP cells.

### Effects of SIRT1 on CEP cell senescence via the p53/p21 pathway under oxidative stress

**Figure 8:**
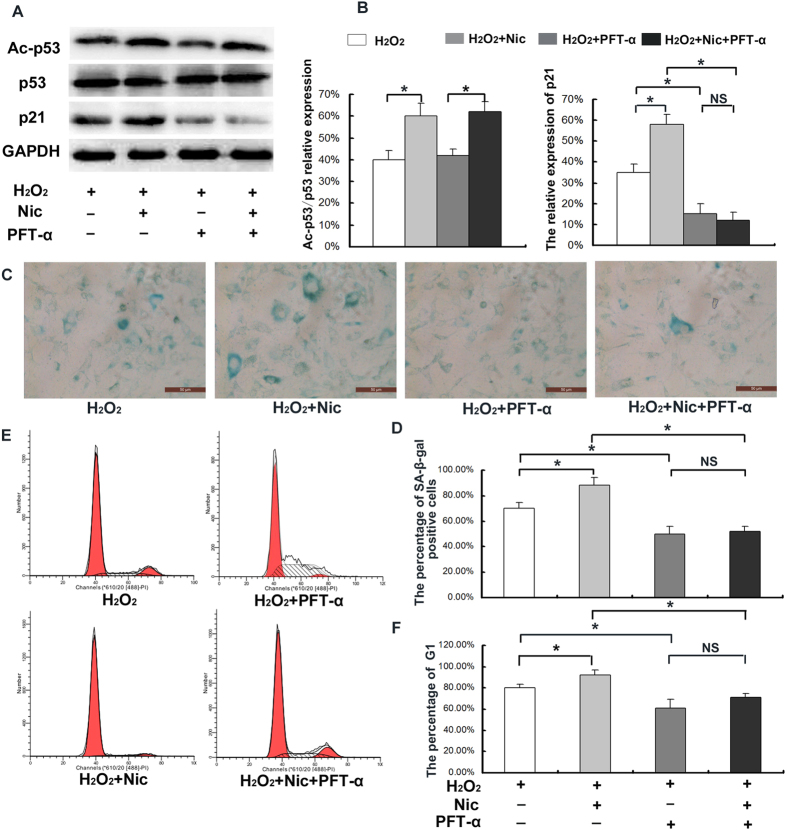
The inhibition of p53 prevented oxidative stress-induced senescence with or without nicotinamide. (**A**) The inhibition of p53 with Pifithrin-α and the levels of acetylated p53 (lys 382) and p21 analyzed via the Western blot method. (**B**) The addition of Pifithrin-α did not affect the level of acetylated p53, but significantly decreased the level of p21 with or without nicotinamide. (**C**) The CEP SA-β-galactosidase staining was performed. (**D**) The addition of Pifithrin-α decreased the percentage of cells positive for SA-β-galactosidase and the staining intensity. (**E**) The CEP cell cycle detected with Flow cytometry. (**F**) The addition of Pifithrin-α decreased the population of G_1_ phase cells. The gels have been run under the same experimental conditions. Three independent experiments were performed on the degenerative human CEP cells from three patients, and the data was denoted in terms of mean ± SD. Nic: nicotinamide. *P < 0.05. NS: *P* > 0.05.

As described above, the overexpression of SIRT1 and inhibition of endogenous SIRT1 activity significantly influenced the p53/p21 pathway and, thereby, CEP cell senescence. Nicotinamide was also used in this study to inhibit endogenous SIRT1 activity in order to further activate the p53/p21 pathway under sublethal oxidative stress. In addition, 100 nM of the p53 inhibitor Pifithrin-α was used to inhibit p53 activity. The results indicated that the addition of Pifithrin-α significantly inhibited p21 protein expression (Fig. 8A,B), and decreased the population of G_1_ phase cells (*p* < 0.05) (Fig. 8E,F) and the percentage of SA-β-Gal-positive cells (blue), and staining intensity (Fig. 8C,D) with or without the addition of nicotinamide. These results further indicated that SIRT1 is capable of alleviating CEP cell senescence via the p53/p21 pathway under H_2_O_2_-induced oxidative stress.

## Discussion

In this study, the cell senescence phenotype was found to be more prominent in the CEP cells obtained from DDD patients than in the CEP cells obtained from age-matched LVF patients. In addition, the results indicated that the p53/p21 pathway plays an important role in the senescence of CEP cells *in vivo* and *vitro*. Furthermore, SIRT1 was found to be capable of alleviating the oxidative stress-induced senescence of degenerative CEP cells in humans via the p53/p21 pathway.

Cellular senescence is induced via two mechanisms^9^. In replicative senescence, telomeres shorten and accumulate in cells as those cells undergo repeated cell divisions^13^. Stress-induced premature senescence is thought to be caused by the presence of various stresses, including reactive oxygen species (ROS)^14^,^15^,^16^, mechanical load^17^, or cytokines, such as interleukin-1^18^. These two types of cell senescence could contribute to disc aging and degeneration in different ways. Replicative senescence appears to occur as a natural process of disc aging, whereas the stress-induced premature senescence of disc cells is thought to be caused by exposure to stress^19^. Similar to articular chondrocytes, adult CEP cells rarely, if ever, divide in normal tissue *in vivo*^20^,^21^. Thus, adult CEP cells unlikely experience telomere shortenening as a result of classical replicative senescence *in vivo*^22^. In addition, degenerated discs have been reported to exhibit higher levels of cell senescence than age-matched non-degenerated discs^23^. According to this data, Extrinsic factors primarily influence cellular senescence in CEP cells. Many studies have shown that aging articular chondrocytes exhibit reductions in proliferative and synthetic capabilities while maintaining the ability to produce pro-inflammatory mediators and matrix degrading enzymes. These senescent secretory phenotypes are likely a result of extrinsic stress-induced senescence caused by oxidative stress rather than intrinsic replicative senescence^24^. Although the role and underlying mechanisms of senescence in cartilage degeneration are unclear, oxidative stress has been proven to be a major mediator of senescence^25^. ROS resulting from excessive mechanical loading, cytokine stimulation, and/or oxidative stress could contribute to DNA damage and subsequent chondrocyte senescence^25^,^26^.

Cellular senescence is induced via two major signaling pathways, including the p38 MAPK/p16^INK4a^ pathway and the p53/p21^cip^ pathway^9^. The results of this study indicated that a higher number of p53- and p21-positive cells were present in the degenerative CEP samples obtained from DDD patients than in the CEP samples obtained from patients with LVF. In addition, oxidative stress induced by H_2_O_2_ activated the p53/p21^cip^ pathway, resulting in CEP cell senescence. Chondrocytes obtained from patients with osteoarthritis (OA) have also been reported to exhibit higher levels of senescence marker p16 than chondrocytes obtained from age-matched normal tissue^27^. However, the CEP cells obtained from LVF and DDD patients did not exhibit a significantly different number of p16-positive cells or evidence of p38 MAPK/p16^INK4a^ pathway activation when cellular senescence was induced by H_2_O_2_ *in vitro*. Furthermore, the p53/p21^cip^ pathway was found to play a more important role in the senescence of CEP cells *in vivo* and *in vitro* than the p38 MAPK/p16^INK4a^ pathway. These results corresponded with the results of a previous study concerning the roles of different pathways in NP cell senescence^28^.

Protein p53 is activated via posttranslational modifications in response to various stress signals^29^. Acetylation of p53 by the p300/CBP transcriptional coactivator occurs in response to the presence of several activators, including UV irradiation, hypoxia, hydrogen peroxide, and the antineoplastic DNA-damaging agents camptothecin and cisplatin^30^. SIRT1 is an NAD^+^-dependent deacetylase that belongs to the sirtuin family of proteins and antagonizes p53-mediated senescence^31^. The role of SIRT1 in CEP degeneration is not well understood. The results of this study indicated that the CEP samples obtained from DDD patients exhibited a significantly lower number of SIRT1-positive cells than the CEP samples obtained from LVF patients. The overexpression of SIRT1 inhibits the p53/p21^cip^ pathway and CEP cell senescence in oxidative stress. In contrast, the inhibition of SIRT1 activity activates the p53/p21^cip^ pathway and, thereby, CEP cell senescence by increasing the levels of acetylated p53. These results indicated that SIRT1 specifically associates with and deacetylates p53, resulting in the negative regulation of p53-mediated transcriptional activation, especially the p53-mediation transcriptional activation of p21, in oxidative stress. Smith^32^ reported that the deacetylation of p53 by SIRT1 prevents cellular senescence and apoptosis resulting from damage to DNA and stress in Mouse Embryonic Fibroblasts (MEFs). SIRT1 promotes cell survival by improving cellular stress tolerance via the down-regulation of p53 transcriptional activity^23^. However, low levels of SIRT1 expression or activity may reduce the ability of CEP cells to overcome adverse conditions and, thereby, accelerate CEP degeneration. Thus, the up-regulation of SIRT1 could improve the abilities of chondrocytes to cope with unfavorable growth conditions^23^. Many studies have also shown that SIRT1 regulates the aging process by stimulating the expression of antioxidants and inhibiting inflammatory responses^33^,^34^. Further studies concerning these effects are needed.

In conclusion, mature IVDs are known to rely almost entirely on the diffusion of solutes across their CEPs for nutrition. Thus, CEPs play an important role in the physiological function of IVDs. Cell senescence induced by stress may contribute to CEP degeneration and, thereby, disrupts physiological function. The results of this study indicated that the p53/p21^cip^ pathway plays an integral role in the senescence of CEP cells *in vivo* and *in vitro* and that SIRT1 is capable of alleviating the oxidative stress-induced senescence of degenerative human CEP cells through the p53/p21^cip^ pathway. This information could be used to further investigate CEP and even IVD degeneration.

## Materials and Methods

### Patients and tissue sources

The subjects of this study were lumbar spine surgery patients admitted to the First Affiliated Hospital of Chongqing Medical University in Chongqing, China from February 2015 to August 2015. Degenerative CEP samples were donated by a total of 29 patients (mean age, 43; age range, 33–50; Pfirrmann, IV–V; 15 males, 14 females) with DDD during discectomy and intervertebral fusion surgery. In addition, age-matched mild-degenerated CEP samples were donated by a total of 5 patients (mean age, 42; age range, 34–48; Pfirrmann, I–II; 3 males, 2 females) with lumbar vertebral fractures (LVF) undergoing posterior discectomy, spinal fusion, decompression, and stability procedures within 24 hours of trauma. These patients, who did not have a documented clinical history of lower back pain (LBP), were used as the control group (Fig. 1A). The CEP samples were extracted in an operation room and delivered to the laboratory within 30 minutes of harvest. A complete culture medium with serum at 4 °C was used as the transport medium. The CEP samples were carefully isolated from the IVD tissue using a scalpel under sterile conditions (Fig. 1B).

An MRI scan of the spine of each patient was performed. The level of disc degeneration was graded according to the Pfirrmann classification^35^. The degenerative grades of the patients are described above.

This study was approved by the ethics committee of Chongqing Medical University, and informed consent was obtained from all of the patients. The methods were carried out in accordance with the approved guidelines.

### Hematoxylin and eosin staining

The retrieved tissues were fixed in 4% paraformaldehyde, decalcified, and embedded in paraffin. The CEP tissues were sectioned from the side of nucleus pulposus (NP) or annulus fibrosus (AF) to bone side via length cutting (4 μm). Serial sections of the embedded specimens were stained with hematoxylin and eosin, this technique was supported by the Tissue Embryology Department of Chongqing Medical University. The resulting stains were photographed under a microscope (Leica, Germany).

### Immunohistochemical analysis

CEP samples were fixed in 4% paraformaldehyde, decalcified, and embedded in paraffin, and then were sectioned from the side of nucleus pulposus (NP) or annulus fibrosus (AF) to bone side via length cutting (4 μm). This technique was supported by the Tissue Embryology Department of Chongqing Medical University. The sections were deparaffinized with xylene and rehydrated using graded ethanol and distilled water. Next, the sections were treated with 3% H_2_O_2_ for 15 minutes at room temperature to eliminate endogenous peroxidasesactivity, incubated with trypsin for 30 minutes at 37 °C to retrieve the antigen, and blocked with normal goat serum for 15 minutes at room temperature. Then, the sections were incubated with rabbit anti-SIRT1 (Abcam, USA, 1:200), rabbit anti-p53 (Cell signal, USA, 1:200), rabbit anti-p21 (Cell signal, USA, 1:200) and rabbit anti-p16 (Ruiying Biological, China, 1:150) primary antibodies overnight at 4 °C. The sections were then incubated with the secondary antibody goat anti-rabbit IgG-HRP. The resulting sections were photographed under a microscope (Leica, Germany). Five fields which distributed in the whole section were counted (400X), and the average positive rate was counted. Three sections were assessed per patient, and three patients were assessed per group (DDD and LVF).

### Cell isolation and cultures

Human CEP samples were separated from the degenerative IVD tissue microscopically according to their different macroscopic morphologies using a scalpel under sterile conditions. Next, the samples were washed twice in a phosphate-buffered saline solution (PBS). The resulting samples were minced, and the matrix was digested for 30 minutes at 37 °C in 0.25% trypsin solution. Next, the samples were incubated overnight at 37 °C in 0.15% type II collagenase. The isolated cells were filtered through a 200-μm filter and resuspended in Dulbecco’s modified Eagle’s medium and Ham’s F-12 medium (DMEM/F12, 1:1) containing 15% fetal bovine serum (FBS) and no antibiotics. The monolayer cell cultures were maintained in a 5% CO_2_ 95% air incubator at 37 °C. All of the experiments were conducted with passage-two human CEP cells. Passage cells were seeded at density of 2.5 × 10^6^/T-25 flasks.

### Infection with Ad-SIRT1, Ad-GFP, and chemicals

Ad-SIRT1 and Ad-GFP were generated using AdEasy technology (Hanbio, china). The CEP cells infected with Ad-SIRT1 exhibited increased SIRT1 expression. CEP cells infected with Ad-GFP were used as the control group. Both the Ad-SIRT1- and Ad-GFP-infected cells expressed green fluorescent protein (GFP), which was used to monitor the infection efficiency via fluorescence microscopy. Nicotinamide (Sigma, USA) dissolved in PBS to a concentration of 12 mM was used to inhibit SIRT1 activity, was dissolved in PBS. In addition, 150 μM H_2_O_2_ was used to induce sublethal oxidative stress^36^. Furthermore, Pifithrin-α (Sigma, USA) dissolved in dimethyl sulfoxide (DMSO) to a concentration of 100 nM was used to inhibit p53 activity^37^.

### Cell treatments

Human CEP cells were seeded at density of 2.5 × 10^6^/T-25 flasks, or were seeded at a density of 1 × 10^6^ /well in 6-well plates. First, Ad-SIRT1 and Ad-GFP were used to infect the cells for 48 hours, or the cells were incubated with 12 mM nicotinamide for 24 hours. They were subsequently incubated with 150 μM H_2_O_2_ for 1 hour. Then, the H_2_O_2_ was removed from the culture, and the cells were incubated in fresh culture medium for 24 hours. The cells were co-incubated with or without Pifithrin-α for 72 hours during the treatment process to a concentration of 100 nM^37^, and then were collected to analyse the difference.

### Flow cytometry

The cell cycle and cell apoptosis were detected by flow cytometry. Following the cell treatments, CEP cells were collected (1 × 10^6^/group), washed twice with PBS, and fixed in 75% ethyl alcohol. The cells in different cycles including G_1_, G_2_ and S were counted and represented as a percentage of the total cell count. In addition, the apoptotic CEP cells were detected using Annexin V/PI double-staining by flow cytometry. The cells were harvested and washed twice with PBS. The early apoptotic cells contained Annexin V+/PI−, the late apoptotic cells contained Annexin V+/PI+, and the normal cells contained Annexin V−/PI−. The early- and late-stage apoptotic cells were counted, and the results were expressed as a percentage of the total cell count. These techniques were supported by the Life Science Department of Chongqing Medical University.

### Senescence-associated β-galactosidase staining

Passage-2 human CEP cells (1 × 10^6^/well/group) obtained from the LVF and DDD patients were cultured in 6-well plates (Thermo Fisher Scientific, USA) for 24 hours, fixed with fixative solution, and subjected to senescence-associated β-galactosidase (SA-β-Gal) staining. The staining process was performed according to the overnight instructions presented in the SA-β-Gal kit (Beyotime, China). Three technical replication were carried out in one patient, and three patients were included in the study. The degenerative human CEP cells were also treated according to this process. The cells were then photographed under a microscope in order to detect any SA-β-Gal activity (Leica, Germany). Five fields which distributed in the whole well were counted (400X), and the average positive rate was counted. Three wells were assessed per group for one patient cells, and cells from three patients were assessed.

### RNA isolation and Real-time PCR

Human CEP cells were seeded at a density of 1 × 10^6^/well in 6-well plates (Fisher, USA) containing DMEM/F12 supplemented with 15% FBS following the different treatments described above. The total RNA was extracted using Trizol reagent (Invitrogen, USA). An iScript cDNA synthesis kit (Bio-Rad, USA) was used to generate cDNA templates from the total RNA through reverse transcription (RT). The first-strand cDNA products were further diluted and used as PCR templates. The PCR primers (Table 1) were designed using Primer 3.0 (ABI Coperation, USA) to amplify the genes of interest. The cDNA was amplified in a 10-μl PCR mix containing 5 μl of SYBR Green Super Mixture (Bio-Rad). The resulting solution was subjected to real-time PCR for 3 minutes at 95 °C using a CFX-Connect Real-Time PCR system (Bio-Rad), followed by 40 cycles at 95 °C for 10 seconds and 58 °C for 5 seconds. The efficiency and specificity values of each primer set were confirmed by comparing the standard curves of the threshold cycle (C_t_) values to the RNA serial dilutions and melting profile evaluations. The C_t_ values were normalized using GAPDH in order to manage any cDNA quantification differences. The results were reported as relative expression levels. Gene expression data was normalized to the control (value = 1) for all genes.

### Protein isolation and Western blotting analysis

CEP cells were seeded at a density of 2.5 × 10^6^/T-25 flasks (Thermo Fisher Scientific, USA), cells containing DMEM/F12 supplemented with 15% FBS following the different treatments described above. The cells were collected with a Lysis Buffer (Beyotime, China). The cleared total cell lysate was denatured via boiling and resolved via 10% sodium dodecyl sulfate polyacrylamide gel electrophoresis. After electrophoretic separation, the proteins were transferred to a polyvinylidene fluoride membrane (Bio-Rad). The membrane was blocked with 5% skim milk for 1 hour at room temperature and probed overnight with anti-SIRT1(Abcam, USA, 1:1000), anti-Ac-histone H_3_ (Ruiying Biological, China, 1:600), anti-p53 (Cell signal, USA, 1:1000), anti-Ac-p53 (lys382) (Cell signal, USA, 1:1000), anti-p21(Cell signal, USA, 1:1000), anti-p16 (Ruiying Biological, China, 1:600), anti-p38 (Ruiying Biological, China, 1:600), anti-phos-p38 (Ruiying Biological, China, 1:600) and anti-GAPDH (Beyotime, China; 1:1000) at 4 °C. After five washes, the blots were incubated with a goat anti-rabbit or rabbit anti-mouse peroxidase-conjugated secondary antibody (Beyotime, China; 1:1000) for 2 hours at room temperature. The blots were displayed in Immobilon Western Chemiluminescent HRP Substrate (Millpore, USA). The protein expression levels were compared to those of GAPDH using Quantity One software (Bio-Rad, Quantity One 4.6.2).

### Statistical analysis

Each of the quantitative assay were technically replicated three times using the same donor tissues/cells, and all of the results were validated with tissues/cells from at least three different donors. The data was expressed in terms of mean ± standard deviation (SD). Any statistically significant differences among the samples were assessed using a one-way variance analysis (SPSS coperation, SPSS 17.0), in which a *p*-value less than 0.05 (*p* < 0.05) was considered statistically significant.

## Additional Information

**How to cite this article**: Zhou, N. *et al.* SIRT1 alleviates senescence of degenerative human intervertebral disc cartilage endo-plate cells via the p53/p21 pathway. *Sci. Rep.* **6**, 22628; doi: 10.1038/srep22628 (2016).

## Figures and Tables

**Table 1:** Oligonucleotide primers utilized for Realtime-PCR amplification.

Garget	Forword primer sequence	Reverse primer sequence	Base pairs
SIRT1	5′-CTGCCTGGATCCCCTTAGTTTTG-3′	5′-GGGCCTGTTGCTCTCCTCATTAA-3′	212 bp
MMP13	5′-GCGTCATGCCAGCAAATTC-3′	5′-TCCCCTACCCCGCACTTC-3′	110 bp
Col2A1	5′-CCGGCACTCCTGGCACTGATG-3′	5′-GGGGGCCAATGGGACCTGTC-3′	216 bp
p53	5′-CCGGCGCACAGAGGAAGAGA-3′	5′-TGGGGAGAGGAGCTGGTGTTGT-3′	108 bp
p21	5′-CCGCCCCCTCCTCTAGCTGT-3′	5′-CCCCCATCATATACCCCTAACACA-3′	98 bp
p38	5′-GGCCGAGCTGTTGACTGGAAG-3′	5′-GCTTGGGCCGCTGTAATTCTCT-3’	267 bp
p16	5′-CCCCGATTGAAAGAACCAGAGA-3′	5′-ACGGTAGTGGGGGAAGGCATAT-3′	190 bp
GAPDH	5′- CTTTGGTATCGTGGAAGGACTC -3′	5′-GTAGAGGCAGGGGATGATGTTCT-3′	133 bp
